# The Role of Immunotherapy or Immuno-Chemotherapy in Non-Small Cell Lung Cancer: A Comprehensive Review

**DOI:** 10.3390/cancers15092476

**Published:** 2023-04-26

**Authors:** Shehab Mohamed, Luca Bertolaccini, Domenico Galetta, Francesco Petrella, Monica Casiraghi, Filippo de Marinis, Lorenzo Spaggiari

**Affiliations:** 1 Department of Thoracic Surgery, IEO, European Institute of Oncology IRCCS, 20141 Milan, Italy; 2 Department of Oncology and Hemato-Oncology, University of Milan, 20122 Milan, Italy; 3 Department of Thoracic Oncology, IEO, European Institute of Oncology IRCCS, 20141 Milan, Italy

**Keywords:** lung cancer, immunotherapy, thoracic surgery, review

## Abstract

**Simple Summary:**

Surgical resections remain the gold standard for early stages non-small-cell carcinoma (NSCLC) and may be considered for locally advanced tumors. Medical treatment has changed drastically in recent years, especially for advanced stages, for which the development of immunotherapy and molecular targeted therapy significantly increased survival and quality of life. The addition of radical surgical resection following immunotherapy or immuno-chemotherapy is feasible and safe with low surgical-related mortality and morbidity in selected patients with initially unresectable NSCLC.

**Abstract:**

Many new treatment modalities for non-small-cell carcinoma (NSCLC) have been described in the last two decades. Surgical resections remain the gold standard for early stages and may be considered for locally advanced tumors. Medical treatment has changed drastically in recent years, especially for advanced stages, for which the development of immunotherapy and molecular targeted therapy significantly increased survival and quality of life. The addition of radical surgical resection following immunotherapy or immuno-chemotherapy is feasible and safe with low surgical-related mortality and morbidity in selected patients with initially unresectable NSCLC. However, data from multiple ongoing trials with overall survival as the primary endpoint should be awaited before this strategy is introduced into the standard of care.

## 1. Introduction

Lung cancer is the leading cause of cancer death worldwide. Many new treatment modalities for non-small-cell carcinoma (NSCLC) have been described in the last two decades, introducing thoracic surgery to a multimodality approach [1]. Surgical resections remain the gold standard for early stages (I-II) and are considered in a multidisciplinary approach for stage IIIA. Medical treatment has changed drastically in recent years, especially for advanced stages (IIIB-IV). For unresectable or metastatic diseases, the development of immunotherapy and molecular targeted therapy significantly increased survival and quality of life in lung cancer patients.

Early-stage NSCLC is defined as localized cancer and refers to stages I, II, and IIIA as described by the 8th edition of TNM [2]. Locally advanced tumors include those with direct invasions, such as Pancoast’s tumors, chest wall infiltrating neoplasia, and tumors with invasion of the main bronchus, the carina, or the pulmonary artery, which require extended pulmonary resections and complex reconstructions of the chest wall, airways, or vessels [3,4].

However, locally advanced tumors also include those with a mediastinal lymph node involvement defining a very heterogeneous group of patients (stage IIIA-IIIB). For locally advanced tumors, surgery may be considered. However, optimal therapeutic management requires an interdisciplinary approach in order to evaluate the extension of the disease at the diagnosis, the patient’s comorbidities and the performance status before the operation, surgical operability, and a systemic induction treatment (also referred to as neo-adjuvant treatment) when indicated for the disease stage, including its potentially toxic effects. In this case, to improve long-term outcomes, the treatment could include platinum-based chemotherapy and, in selected cases, a specific radiotherapy program to reduce tumor size and lymph node involvement before complete resection. Several studies compared survival and outcomes between definitive chemo-radiotherapy and surgery after induction treatment (chemotherapy or chemo-radiotherapy) [5].

Chemotherapy drugs, especially platinum-based compounds, are associated with side effects [6]. In this view, numerous molecular mutations in cancer biology have been searched and identified in NSCLC patients to develop new therapeutic strategies with lesser adverse reactions and better oncological outcomes in recent years. Specific molecular mutations may classify new therapies as tyrosine kinase inhibitors (target therapy) or immune checkpoint inhibitors (immunotherapy).

Regarding tyrosine kinase inhibitors (TKI), the most commonly identified targets in the adenocarcinoma setting are activating gene K-RAS and EGFR [7], re-arranged genes ALK and ROS-1 [8,9], and many others. Though molecularly targeted therapies in the neo-adjuvant setting are associated with a decrease in the risk of recurrence and an increase in the mediastinal downstaging rate, they are not associated with a complete pathological response [10].

In the last decade, different retrospective studies have shown significant outcome changes in previously unresectable diseases treated with tyrosine kinase inhibitors followed by lung resections for residual disease when feasible [11,12]. Based on these promising results, the latest National Association of Medical Oncology guidelines confirmed that all patients with non-squamous histology or mixed and young non-smoker patients with squamous histology should be tested for ALK and EGFR [13,14].

Erlotinib safety, tolerability, and pathological responses were evaluated in patients with EGFR-mutated NSCLC in a phase II study which showed encouraging results [15]. A reasonable response rate was found on using Lorlatinib and Crizotinib as neoadjuvant therapy in a significant phase III trial in patients with advanced rearranged-ALK NSCLC [16].

Regarding the role of the immune checkpoint inhibitors, Durvalumab has been established in the PACIFIC trial as the standard of care for stage III unresectable NSCLC patients as consolidation therapy after concurrent chemoradiation [17]. This role has been questioned in patients affected by EGFR-mutated NSCLC after definitive chemo-radiotherapy [18].

Many trials are studying the effects of tyrosine kinase inhibitors in this group of patients as consolidation therapy; for example, Osimertinib safety and efficacy are being assessed in a currently ongoing global phase 3 trial following concurrent or sequential chemoradiation in a patient with stage III (A/B/C) unresectable NSCLC and EGFR positive [19]. Conversely, immunotherapy may be used as a neoadjuvant or definitive therapy, as already reported in several clinical trials for resectable NSCLC and unresectable NSCLC.

## 2. Neoadjuvant Immunotherapy or Immuno-Chemotherapy in Resectable NSCLC

Administering immune checkpoint inhibitors alone or in combination with chemotherapy and followed by surgical resections can benefit patients in terms of OS and DFS. Despite surgical resection being the standard of care for early-stage NSCLC, micrometastases, and isolated tumor cells are very challenging to detect by current technologies. Immune checkpoint inhibitors with or without chemotherapy combined with surgery can lower the probability of recurrence eradicating the micrometastases (Table 1 and Table 2).

### 2.1. Neoadjuvant Immunotherapy

Forde et al. in 2018, in one of the first single-arm clinical trials, showed the potential benefit of neoadjuvant Nivolumab followed by lung resection in a group of 21 patients with resectable NSCLC ranging from stage IB to IIIA. In his study, major pathological response and complete pathological response were achieved in nine and two patients, respectively [35]. After this study, other large trials have been reported, such as NEOSTAR, which assigned patients randomly to be treated with neoadjuvant Nivolumab alone or in combination with Ipilimumab; this phase II study analyzed resectable stage I-IIIA NSCLC patients treated with three cycles of Nivolumab or in combination to one dose of Ipilimumab. The major pathological response rate was achieved in 38% of patients after the combined therapy, whereas a 22% rate was reported in the monotherapy arm. Similar results have been reported for a complete pathological response of 29% and 9%, respectively [20].

LCMC3 (Lung Cancer Mutation Consortium) trial, the most significant phase II trial reported to date, considered 181 patients with pathologically documented stage IB-IIIB resectable NSCLC, including T3N2 or T4 (by size criteria, not by mediastinal invasion), who received before the surgery two cycles of Atezolizumab, and for those who showed clinical response, the treatment was continued for up to 1 year. In this group, 144 patients showed a major and complete pathological response in 20% and 7% of the cases, respectively. PD-L1 positivity and higher tumor mutation burden were correlated to a more significant pathological response [21].

Other trials have been reported analyzing the role of neoadjuvant single-agent immunotherapy in resectable NSCLC. The role of Sintilimab was evaluated in 40 enrolled patients with stage IA-IIIB by Gao et al., 37 of whom underwent surgery. In this study, 40.5% achieved MPR and 16.2% pCR [22].

Pembrolizumab efficacy and safety outcomes were evaluated in the NEOMUN trial. Fifteen patients with clinical-stage II-IIIA resectable NSCLC received two cycles of Pembrolizumab before surgery. Of the enrolled patients, 33% had relevant adverse effects. Two patients had MPR, and two other patients had pCR [23].

Atezolizumab in the neoadjuvant setting was analyzed in the PRINCEPS trial in 30 patients with clinical IA (≥2 cm)-IIIA NSCLC. Among these, 29 had R0 resection. No pCR was reported, while 14% had MPR, and 41% had pathological response ≥ 50% [24].

IONESCO is a phase II trial that analyzed the role of Durvalumab prior to surgical resection in 50 patients with stage IB (tumor ≥ 4 cm)-IIIA resectable NSCLC. Among these patients, 90% achieved R0, with an MPR of 18.6% and pCR of 7%. The enrolment in this study was stopped due to 9% of death at 90 days, attributed by the authors not to Durvalumab toxicities but to pre-existing comorbidities [25].

Tong et al. published in February 2022 a study that examines the safety and efficacy of neoadjuvant Pembrolizumab in stage IB to IIIA NSCLC. Thirty-five patients were enrolled, thirty received two cycles of Pembrolizumab, and twenty-five underwent surgery (72% minimally invasive surgical approach) followed by a complete resection in 88% of the cases. In this case series, neoadjuvant Immune Checkpoint Inhibitors (ICIs) seem safe and tolerated, and no association with increased toxicity, delays before surgery, or additional surgical morbidity was reported [26].

The role of radiotherapy in combination with immunotherapy as an immunomodulator has been assessed by Altorki et al. His group examined the combination of neoadjuvant Durvalumab with stereotactic body radiotherapy in patients with early-stage lung cancer (I-IIIA). This randomized, single-center phase II trial evaluates the immunomodulatory activity of radiotherapy in patients with early-stage NSCLC enhancing the antitumor immune response associated with the anti-PD-L1 antibody Durvalumab. In this trial, stereotactic body radiotherapy might be a potent immunomodulator in advanced NSCLC, is well tolerated, and is associated with a higher major pathological response rate than the Durvalumab monotherapy group [27] (Table 1).

### 2.2. Neoadjuvant Immuno-Chemotherapy

Several studies have demonstrated the superiority in oncological outcomes of immunochemotherapy compared to chemotherapy alone in the neoadjuvant setting from early stage to locally advanced NSCLC patients (Table 2).

Shu et al. published a phase II multicenter study of Atezolizumab and chemotherapy (carboplatin plus nab-paclitaxel) for up to four cycles in 30 patients affected by resectable NSCLC stage IB-IIIA and smoking history. Twenty-three patients had stage IIIA disease, and 87% underwent complete tumor resection [29].

In the NADIM II trial, which enrolled 86 patients with stage IIIA or stage IIIB disease and no known EGFR or ALK alterations, the pathologic complete response rate was 36.8% in 57 patients who received Nivolumab plus Paclitaxel and Carboplatin against 6.9% rate observed among the 29 patients treated with neoadjuvant chemotherapy alone. Provencio et al. also reported a higher major pathologic response in the first group, 52.6% vs. 13.8%, respectively. This trial confirms the superiority of the neoadjuvant Nivolumab plus chemotherapy combinations in patients with resectable stage IIIA-B NSCLC. These preliminary results were presented at the 2022 American Society of Clinical Oncology (ASCO) annual meeting in Chicago and the International Association for the Study of Lung Cancer (IASLC) 2022 World Conference on Lung Cancer (WCLC) in Vienna. The immunotherapy combination maintained a tolerable safety profile and a moderate increase in grade 3–4 toxicity [30].

Another phase III trial named CheckMate 816 compares three cycles of neoadjuvant Nivolumab plus platinum-doublet chemotherapy with the control arm alone. According to the seventh edition staging, this study enrolled 358 patients with stage IB-II-IIIA NSCLC. The pathologic complete response and major pathologic response were higher in patients treated with immunochemotherapy than those treated with chemotherapy, 24% vs. 2% and 37% vs. 9%, respectively. Spicer presented the surgical outcomes of this study at the ASCO Annual Meeting in 2021. These results revealed that a higher number of patients underwent surgery with no interference in feasibility or timing in the study group compared to the standard group (83% vs. 75%). More patients underwent lobectomies, and fewer underwent pneumonectomies compared to the standard group who received chemotherapy alone (77% vs. 61% and 17% vs. 25%).

Moreover, no increase in toxicity of postoperative complications was reported in the study group with the addition of ICIs [31]. Following these results, the FDA approved Nivolumab and platinum-based chemotherapy in the neoadjuvant setting for NSCLC [36].

In 2022, the Checkmate 816 results were published. More prolonged event-free survival (HR 0.63), a higher percentage of pCR (24% vs. 2.2%), better OS, time to distant metastases, MPR, and radiographic downstaging were reported for Nivolumab plus chemotherapy compared to chemotherapy alone as neoadjuvant treatment in resectable NSCLC. In addition, no increase in adverse effects or decrease in the feasibility of surgery was reported in the study group [35].

Several other phases III neoadjuvant chemotherapy plus immune checkpoint inhibitors studies are ongoing, such as:

- KEYNOTE 671 evaluates the safety and efficacy of Pembrolizumab in combination with platinum doublet neoadjuvant chemotherapy before surgery, followed by Pembrolizumab alone after surgery in resectable stage II, IIIA, and resectable IIIB (T3-4N2) NSCLC [32].
- AEGEAN assesses the activity of Durvalumab and chemotherapy in stage II-III NSCLC patients, administered before surgery, compared with placebo and chemotherapy administered before surgery in terms of complete pathological response, with encouraging preliminary results. A statistically significant improvement in major pathologic response was observed [33].
- CheckMate 77T trial evaluates neoadjuvant Nivolumab in combination with chemotherapy followed by adjuvant Nivolumab in resectable stage IIA–IIIB (T3N2 only) NSCLC patients. The primary endpoint of the study is EFS [20].
- IMPOWER 030 study evaluates the safety and efficacy of Atezolizumab in combination with platinum-based chemotherapy as a neoadjuvant treatment in resectable stage II-III NSCLC patients [34].

Most studies demonstrate the efficacy and tolerability of immune checkpoint inhibitors in the unresectable NSCLC in the adjuvant setting.

## 3. Adjuvant Immunotherapy

Chemotherapy has been the standard of care for patients with NSCLC as adjuvant therapy, improving the 5-year survival rates by only 4–5%.

Adjuvant therapy plays an essential role in preventing recurrence and eliminating micrometastases. To date, new targeted therapies have been described that showed better DFS than chemotherapy, such as Osimertinib [37], Gefitinib [38], or Erlotinib [39].

Wu et al. reported in ADAURA that 90% of patients with stage II to IIIA were alive at 24 months in the Osimertinib group and only 44% in the placebo group.

In recent years, ICIs have become more and more utilized for unresectable locally advanced or metastatic stages with very promising results, as described in the following paragraphs. Based on these results, several new trials are studying the efficacy of ICIs also in early-stage NSCLC patients.

Most of the ongoing clinical trials in this field are evaluating the benefits of ICIs associated with chemotherapy as adjuvant treatment for resectable NSCLC (Stage IB-IIIA and resectable IIIB) (Table 3):

- IMpower 010: open-label phase III study with a sample size of 1280 patients from 22 countries. This trial includes EGFR mutations and ALK rearrangements. A total of 1005 patients were randomized to receive adjuvant Atezolizumab (507) or best supportive care (498). In patients with stage II-IIIA NSCLC and an expression of PD-L1 ≥ 1%, the patients who had disease progression were 35% of patients receiving Atezolizumab and 46% of patients receiving best supportive care, reducing the risk of recurrence by 34% (HR = 0.66; 95% CI: 0.50–0.88) [40]. Due to these promising results, the FDA approved Atezolizumab as adjuvant monotherapy in patients with PD-L1 positive in October 2021 [41]. At the 2022 European Lung Cancer Congress (ELCC 2022), Felip et al. presented the updated preliminary results for DFS in patients with PD-L1 ≥ 50% stage II-IIIA NSCLC, with or without EGFR mutations or ALK rearrangements. In patients with EGFR mutations or ALK rearrangements, the 3-year DFS rates were 73.8% in the Atezolizumab group compared to 48.6% in the control group. In patients without EGFR mutations or ALK rearrangements, the 3-year DFS was 75.1% in the Atezolizumab group and 50.4% in the control group [42]. Adjuvant Atezolizumab was associated with a 22% recurrence rate compared to 44%, a median recurrence of 18.1 months vs. 10.1 months, and a lower rate of distant metastasis of 9% vs. 26%. The analyses of OS were presented at the 2022 World Conference on Lung Cancer (WCLC 2022). In the PD-L1 ≥ 1% patients, the Atezolizumab group showed a 5-year OS rate of 76.8% vs. 67.5% in the control group. Furthermore, in the PD-L1 ≥ 50% of patients, the 5-year OS rates were 84.8% in the Atezolizumab group compared to 67.5% in the control group [43].
- KEYNOTE-091: phase III trial with a sample size of 1177 patients with stage IB-IIIA. In this study, patients after adjuvant chemotherapy are randomized to receive adjuvant Pembrolizumab or placebo for one year [44]. Preliminary results showed an improved DFS of 53.6 months in the Pembrolizumab group compared to 42.0 months in the placebo group. At the ESMO Congress 2022, Peters et al. reported based on the PD-L1 status, the 3-year median DFS which was 65.9% vs. 57.6% in PD-L1 ≥ 50%, 54.6% vs. 44.8% in PD-L1 1–49% and 55.5% vs. 48.8% in PD-L1 < 1% [45].
- BR31/IFCT1401: phase III trial with a sample size of 1415 patients with a stage IB-IIIA NSCLC. After adjuvant chemotherapy, patients are randomized to receive Durvalumab or a placebo for one year. The outcome studied is DFS in patients with PD-L1 ≥ 25% without EGFR mutations or ALK rearrangements [46].
- ANVIL, ALCHEMIST Chemo-IO, MERMAID-1, MERMAID-2, NADIM-ADJUVANT, and LungMate-008 are ongoing trials studying efficacy, the DFS and the OS of adjuvant ICIs (Nivolumab, Pembrolizumab, Durvalumab, Toripalimab) in patients with resectable NSCLC (see Table 3 for details) [47,48,49,50,51,52].

As described in the NCCN guidelines, the recommended duration of Atezolizumab as adjuvant therapy is one year [53]. In the previously reported ongoing trials, the adjuvant ICIs treatment ranges from 6 months to 2 years. Future results will better define the efficacy and duration of adjuvant therapy, as well as the predictors of response and the combination of multiple therapies.

**Table 3:** Clinical Trials: Adjuvant Immunotherapy, Immuno-Chemotherapy in resectable NSCLC.

Adjuvant Immunotherapy						
Trial/Study Name	Phase	Patient N	Neoadjuvant Therapy	Patient Population	Outcomes	Safety
IMpower010 [41,42,43]	III	1280	CT ± Atezolizumab	IB-IIIA	PD-L1 ≥ 50%, EGFR+, ALK+: 3-y DFS 73.8% vs. 48.6%. PD-L1 ≥ 50%, EGFR−, ALK−: 3-y DFS 75.1% vs. 50.4%. PD-L1 ≥ 1%: 5-y OS 76.8% vs. 67.5%. PD-L1 ≥ 50%: 5-y OS 84.8% vs. 67.5%.	NR
KEYNOTE-091 [44,45]	III	1177	Pembrolizumab vs. Placebo	IB-IIIA	Median DFS 53.6 months vs. 42.0 months. PD-L1 ≥ 50%: 3-y DFS 65.9% vs. 57.6%. PD-L1 1–49%: 3-y DFS 54.6% vs. 44.8%. PD-L1 < 1%: 3-y DFS 55.5% vs. 48.8%	NR
BR31/IFCT1401 [46]	III	1415	Durvalumab vs. Placebo	IB-IIIA	Not yet	Not yet
ANVIL [47]	III	903	Nivolumab vs. Observation	IB-IIIA	Not yet	Not yet
ALCHEMIST Chemio-IO [48]	III	1210	CT ± Nivolumab	IIA-IIIB	Not yet	Not yet
MERMAID-1 [49]	III	332	CT ± Durvalumab	II-III, MRD	Not yet	Not yet
MERMAID-2 [50]	III	284	Durvalumab vs. Placebo	II-III, MRD	Not yet	Not yet
NADIM-ADJUVANT [51]	III	210	Paclitaxel + Carboplatin ± Nivolumab	IB-IIIA	Not yet	Not yet
LungMate-008 [52]	III	341	CT ± Toripalimab	II-IIIB	Not yet	Not yet

OS: Overall Survival, CT: Platinum-doublet chemotherapy, MRD: Minimal Residual Disease, y: years, NR: Not Reported.

## 4. Immunotherapy or Immuno-Chemotherapy in Unresectable NSCLC

For unresectable NSCLC, several therapeutic strategies have been proposed in the last decade. these treatments could range from local therapies (such as radiotherapy) to systemic therapies (such as standard chemotherapy, target therapy, and immunotherapy), which may be used alone or in association with each other.

### 4.1. Unresectable Stage III NSCLC

One of the most representative studies regarding the association between chemotherapy and radiotherapy was published in 2014. In this view, sequential and concurrent chemotherapy were compared in a group of stage III unresectable NSCLC patients. For sequential therapy, the median Overall survival (OS) and the 5-year survivals were 17.4 months and 17%, respectively, against a median OS of 18.6 months and a 5-year survival rate of 19% for the concurrent chemo-radiotherapy group [54].

Bradley and colleagues described in 2015, in a randomized phase 3 study, concurrent chemotherapy associated with standard-dose versus high-dose radiotherapy in patients with locally advanced stage III unresectable NSCLC. In this study, unfortunately, most of the initially unresectable locally advanced NSCLC patients experienced disease progression, despite definitive treatment [55].

Considering these findings, several efforts have been made to increase the OS and DFS in unresectable NSCLC patients, and in this view, different authors focused mainly on immunotherapy. In 2017, in a large study published in the New England Journal of Medicine (NEJM), Antonia et al. demonstrated a significant improvement in OS and time to distant metastatic disease using chemoradiation followed by 12 months consolidation immunotherapy with Durvalumab in unresectable stage III NSCLC patients, becoming the new standard of care [56]. This randomized international phase III study was defined as PACIFIC TRIAL, and on this base, updated analyses have been made by Spigel et al. in 2022 [57]. A total of 713 patients with stage III unresectable NSCLC, who did not progress with following platinum-based concurrent chemo-radiotherapy, were randomized in two arms; in the first, 476 received Durvalumab IV 10 mg/kg up to 12 months, and in the second arm 237 received placebo IV q2w up to 12 months. The inclusion criteria were WHO performance status 0 or 1, histologically or cytologically documented stage III, unresectable NSCLC who had received at least two cycles of concurrent CRT, which must be completed within 1 to 42 days prior to the first dose. Patients with toxicity grade > 2 (standard terminology criteria for adverse events) or grade > 2 pneumonitis or radiation pneumonitis from prior CRT were excluded. For a patient with a lower grade, the first dose of investigational product could be delayed up to 42 days from the end of chemoradiation therapy. Co-primary endpoints were PFS by blind independent central review and OS. This global study showed promising results, such as an increased OS at five years follow-up by 28% with a Hazard Ratio of 0.72 using Durvalumab. After five years, 42.9% of the patients treated with immunotherapy are still alive. Conversely, PFS is increased with a hazard ratio of 0.55 and a decreased risk of progression under immunotherapy. After five years, one patient over three does not have disease progression. This 5-year data from PACIFIC demonstrated robustly and sustained OS plus durable PFS benefits with Durvalumab in this patient population [57].

All the PD-L1 subgroup patients showed increased OS and DFS when treated with Durvalumab against a placebo. The only doubt about the immunotherapy benefit regards the negative PD-L1 patients. In this latter subgroup, Durvalumab positively impacted PFS but not OS.

These study results have been taken into account by several associations. The European Medicines Agency (EMA) has concluded that patients with stage III NSCLC with PD-L1 < 1% should not receive treatment with Durvalumab [58]. This conclusion raised many concerns among international lung experts, who have stated that this approval decision can create disparities across countries with differential treatment access. Conversely, in the USA, the Food and Drug Administration (FDA) authorized to use of Durvalumab in all patients, even if they are PD-L1 negative [59].

In view of this, the PACIFIC-R, a real-world international and observational study, was presented at the European Society of Medical Oncology (ESMO) congress in 2021 and included 1399 patients from 11 participating countries: France (342), Spain (244), Australia (165), Netherlands (155), Belgium (188), Italy (116), Israel (92), Germany (62), UK (54), Norway (36), and Switzerland (15). This study focused on treatment duration and analysis of PFS in unresectable stage III NSCLC patients treated with Durvalumab after chemo-radiotherapy regardless of tumor PD-L1 expression. The primary endpoints assessed were PFS and OS. An essential inclusion criterion was the non-evidence of progression following platinum-based chemotherapy concurrent or sequential to radiotherapy within the previous 12 weeks. Patients were treated with Durvalumab (10 mg/kg IV q2W), and retrospective data were collected at different time points. The median time to Durvalumab initiation from the end of RT was 56 days. The overall median Durvalumab treatment duration was 335 days. Patients received a median of twenty-two Durvalumab infusions. 76.8% of patients received concurrent chemo-radiotherapy, and 14.6% received sequential chemo-radiotherapy. There were no significant differences in baseline characteristics, except for patients older than 70 years old and the longest dimension of the primary tumor. The median PFS in PACIFIC-R was higher than the median PFS reported for the Durvalumab arm of the PACIFIC trial. In this “real-world study,” the median follow-up duration was 23 months, but it is likely overestimated due to several factors:

- 50 early deaths before study enrolment were not counted in the Germany and UK sites.
- RECIST criteria (Response Evaluation Criteria in Solid Tumors) for tumor assessment has been used heterogeneously across different countries.
- In the real world, assessment for disease progression may not occur as frequently and systematically as in other clinical trials. Moreover, the COVID-19 pandemic has also resulted in fewer hospital visits and inconsistent follow-up.

The median PFS in patients PD-L1 > 1% was 22.4% instead of 16.3% in PD-L1 negative. The PFS was 23.7% in stage IIIA and 19.2 months in stage IIIB/C. Once again, the PFS was 25.3 in non-squamous lung cancer patients and 14.7 months in squamous cancer patients. The PFS was 23.7 months in concurrent chemo-radiotherapy vs. 19.4 months in the sequential chemo-radiotherapy subgroup of patients.

The most common adverse effect of Durvalumab was pneumonitis/interstitial lung disease (LID), leading to a permanent discontinuation in 9.5% or a temporary interruption in 5.2% of the cases. The median time to onset pneumonitis/LID from Durvalumab initiation was 2.5 months, and corticosteroid administration was required in 71.3% of the cases [60].

PACIFIC-6 is an ongoing phase II open-label and multicenter study where patients with unresectable stage III NSCLC, who have not progressed following platinum-based sequential chemoradiation therapy, receive Durvalumab 1500 mg IV q4w up to 24 months or until disease progression, with safety and tolerability as primary endpoints. Considering the adverse effects correlated to therapy, 70% were of any grade against 4% of grades 3 and 4. Among the 117 patients, 25 discontinued Durvalumab for side effects, 9 of which were mainly for pneumonitis. As a secondary outcome, PFS was considered in this study. Until now, preliminary results were PFS related to ECOG performance status (PS). Patients classified as ECOG PS 0–1 (*n* = 114) had a PFS of 13.1 months; conversely, patients classified as ECOG PS 2 (*n* = 3) had a PFS of only 3.7 months. Considering the low sample size of the latter group, these results could not be statistically significant [61].

Moreover, PACIFIC 2 is an ongoing phase 3, randomized, double-blind, placebo-controlled, multicenter, global study that is assessing the efficacy and safety of Durvalumab given concurrently with platinum-based CRT (Durvalumab plus standard of care CRT) in patients with locally advanced, unresectable NSCLC (stage III) compared to placebo plus CRT followed by placebo. The estimated study completion date is November 2023 [62].

In synthesis, these first different trials showed an improved OS and PFS in unresectable stage III NSCLC patients treated with Durvalumab after CRT, especially if the tumor is adenocarcinoma at earlier stages, if the PD-L1 is positive and if the CRT is concurrent over sequential chemo-radiotherapy. The rationale of the CRT followed by Durvalumab is that chemoradiation induces tumor antigen release and adaptive immune response, and therefore PD-L1 overexpression leads to immune cell evasion. In this scenario, Durvalumab reverses immune suppression and leads to a systemic antitumor response. Nevertheless, results shared by the PACIFIC trial have been considered improvable, as only approximately 50% of the patients are alive after four years of Durvalumab initiation. In this view, Durvalumab and several other immune checkpoint inhibitors alone or combined with systemic therapy, chemotherapy, and radiotherapy have been studied to improve OS and PFS in NSCLC and SCLC patients.

COAST (Combination Platform Study in Unresectable Stage III NSCLC; NCT03822351) is a global, open-label, randomized, Phase II study of Durvalumab alone or combined with the anti-CD73 mAb Oleclumab or anti-NKG2A mAb Monalizumab as consolidation therapy in patients who have not progressed after concurrent chemo-radiotherapy. This is the first study showing improved outcomes with novel immune-oncology combinations in the PACIFIC setting.

The rationale for using Oleclumab and Monalizumab is that radiotherapy induces CD73 and HLA-E (NKG2A ligand) expression, which inhibits the antitumor immune response. Oleclumab inhibits CD73 to reduce extracellular adenosine production, thereby promoting antitumor immunity, and it has been shown to have a durable response and manageable safety when combined with Durvalumab in a phase I study. Monalizumab reduces the inhibition of NK and CD8+ T cells by blocking NKG2A.

Between January 2019 and July 2020, 189 patients were randomized, of whom 66 received Durvalumab, 59 received Durvalumab plus Oleclumab, and 61 received Durvalumab plus Monalizumab. Study results were published in the Journal Clinical of Oncology in 2022, indicating that the combination of Oleclumab or Monalizumab with Durvalumab provides an additional clinical benefit over Durvalumab alone in patients with unresectable, stage III NSCLC without disease progression following cCRT. After a median follow-up of 11.5 months (range: 0.4–23.4; all patients), the objective response rate (ORR) was higher for both the combinations (30% with Durvalumab plus Oleclumab and 35.5% with Durvalumab plus Monalizumab) compared to Durvalumab alone (30%).

Both combinations also significantly improved PFS versus Durvalumab alone. PFS benefit with both combinations was observed across various subgroups, including those based on histology, ECOG PS, prior platinum-based CT, and PD-L1 status. Median PFS was 6.3 (95%CI, 3.7–11.2) with Durvalumab, not reached (95%CI, 10.4-NR) with Durvalumab plus Oleclumab and 15.1 months (95%CI, 13.6-NR) with Durvalumab plus Monalizumab. Safety profiles were consistent across arms, with no significant safety signals identified in either combination arm. The incidence of adverse effects for Durvalumab, including pneumonitis, were similar across arms [63].

These findings have led to the phase III PACIFIC-9 study, which is a phase III, double-blind, multicenter international study of Durvalumab plus Oleclumab and Durvalumab plus Monalizumab for the treatment of patients who have not progressed following concurrent chemoradiation treatment for locally advanced, stage III, unresectable NSCLC. This trial started in February 2022 and assessed PFS and OS between these drugs and their safety and tolerability [64].

Another successful combination between immune checkpoint agents has been reported by Cho et al. in the CITYSCAPE clinical trial, which analyzed the effects of Tiragolumab, an anti-T-cell immunoreceptor with Ig and ITIM domains (anti-TIGIT), in combination with Atezolizumab (anti-PD-L1) in chemotherapy-naïve, PD-L1 positive, locally advanced, and metastatic NSCLC. CITYSCAPE is a phase II, randomized, double-blind, placebo-controlled trial, which showed an improved ORR and PFS in the anti-TIGIT with anti-PD-L1 therapy combination with more significant benefit observed in the PD-L1 tumor proportion score (TPS) ≥ 50%. Tiragolumab and Atezolizumab were well-tolerated, with a safety profile similar to placebo plus Atezolizumab [65]. Based on these results, the PACIFIC 8 phase III trial studies the effects of Durvalumab plus anti-TIGIT (Domvanalimab) as sequential therapy in participants with stage III NSCLC who have not progressed following definitive, platinum-based concurrent chemo-radiotherapy. The estimated study completion date is September 2029 [66].

Pembrolizumab, Nivolumab, Ipilimumab, and Durvalumab are being studied alone or in combination with other systemic therapies or radiotherapy in several other phases II and phase III ongoing trials, some of these with already preliminary results (KEYNOTE 799, NICOLAS, CheckMate 73L, BTCRC-LUN16-081, DUART), to evaluate the safety, efficacy, PFS, and OS in unresectable stage III NSCLC patients [67,68,69,70,71,72,73,74,75].

Additional immunomodulation through combination therapy is being explored to improve clinical outcomes in this patient population.

Starting from 2018, retrospective data have shown limited activity of ICI monotherapy in patients with advanced NSCLC with specific driver genomic alterations (dGA). Mazieres et al. reported an overall survival of 13.3 months and a PFS of 2.8 months in the whole cohort of 551 patients. Outcomes for patients with dGA (EGFR, ALK, ROS1) were inferior, so ICI should be considered after exhaustion of other systemic therapies such as targeted or chemotherapies or combined with chemotherapy. With comparable results in 2020, Guisier et al. studied the efficacy of ICI against patients with dGA-NSCLC that seemed close to that observed in unselected patients with NSCLC. [76,77]. Moreover, in another study, preliminary evidence has shown the absence of the benefit of Durvalumab in stage III NSCLC EGFR-mutant population [18].

Riudavets M. et al. presented at the ESMO Congress 2021 preliminary outcome results of Durvalumab as consolidation therapy in patients with stage III unresectable NSCLC with driver genomic alterations, which was then published in January 2022 in the European Journal of Cancer. This multicenter retrospective study has 25 participating centers from Europe, the United States, and Argentina. Out of 323 patients included from April 2015 to October 2020, 43 had at least one dGA (26 KRAS, 8 EGFR, 5 BRAF, 4 ALK). Among these, 13% had negative PD-L1 status. In the overall population, the median follow-up was 18.5 months (95%CI, 16.9–21) with a median PFS of 17.5 months (95%CI, 13.2–24.9) and median OS of 47.0 months (95%CI, 47.0-not reached). Considering the PD-L1 status, no differences in median PFS between the PD-L1 positive and PD-L1 negative population were reported. Moreover, no differences in median PFS between dGA (14.9 months) and non-dGA groups (18 months) were reported [78].

Considering Durvalumab outcomes by molecular subgroups, this study observed differences for Durvalumab as PFS among the different molecular subgroups in stage III unresectable NSCLC; patients with NSCLC with KRAS mutation had better ICI outcomes, in contrast to patients with other driver molecular alterations, such as EGFR mutations, BRAF mutations or ALK rearrangements.

Based on these results, this study showed the limited activity of Durvalumab in stage III unresectable NSCLC patients with EGFR mutation, BRAF mutation, and ALK rearrangement but not for KRAS mutation. In the latter case, patients with this rearrangement should still receive immunotherapy consolidation in multidisciplinary evaluation [78].

These studies show no benefit for immunotherapy (Durvalumab) in dGA-NSCLC patients, except for NSCLC patients harboring KRAS mutation (Table 4).

### 4.2. Metastatic NSCLC without Driver Mutations

Several studies and trials reported the outcomes of anti-PD-1/PD-L1 monotherapy alone or in combination with platinum doublet chemotherapy or cytotoxic T-lymphocyte-associated antigen 4 (CTLA-4) blocking antibody against standard platinum doublet chemotherapy in advanced NSCLC patients (Table 5).

More specifically, in the last decade, different clinical trials evaluated the use of Pembrolizumab as anti-PD-L1 monotherapy compared to the standard-of-care chemotherapy analyzing its OS, PFS, ORR, tolerability, and safety.

The approval of Nivolumab for advanced NSCLC by the FDA was based on two phase III clinical trials, Checkmate 017 and Checkmate 057 [79,80]. The first enrolled 272 patients with advanced squamous cell NSCLC, randomized to receive Nivolumab or Docetaxel. The Median OS was 9.2 months in patients treated with Nivolumab compared to 6.0 months. Conversely, in CheckMate 057, 582 patients with non-squamous NSCLC were enrolled; in this study, the median OS was 12.2 months for patients receiving Nivolumab compared to 9.4 months for patients treated with Docetaxel. Moreover, the survival benefit of Nivolumab compared to Docetaxel was confirmed at a 2-year follow-up. Based on CheckMate 057, PD-L1 expression was not associated with a survival benefit for patients with squamous cell carcinoma but was a predictive biomarker for response to Nivolumab for patients with non-squamous NSCLC [96].

Keynote-001, Keynote-024, and Keynote-042 demonstrated exciting results favoring Pembrolizumab over chemotherapy regardless of PD-L1 expression [81,82,83].

However, in 2011, the first CTLA-4 inhibitor to receive FDA approval was Ipilimumab for patients with metastatic melanoma [97]. Another promising inhibitor still not currently approved by the FDA is Tremelimumab, which is now used in several clinical trials.

Govindan et al. reported the results of a phase III trial on Ipilimumab combined with Paclitaxel and Carboplatin in advanced squamous NSCLC. In this study, no significant OS benefit was demonstrated in the chemoimmunotherapy group compared to chemotherapy alone, and more toxicity was related to treatment with Ipilimumab, resulting in many cases of discontinuation and reduced potential benefit [84].

In this view, advanced NSCLC patients are being studied in several ongoing trials, which now focus on treatment based on CTLA-4 inhibitors in combination with other immune checkpoint inhibitors with or without the addition of chemotherapy.

In contrast, the combination of Tremelimumab and Durvalumab compared to chemotherapy was evaluated in the phase III MYSTIC and NEPTUNE trials. Both trials showed no benefit in OS or PFS with the combination of these two therapies.

The only difference in results was the improvements in Rizvi et al. study of OS, PFS, and ORR for this combination versus chemotherapy in specific patients with blood-based TMB measurement (bTMB) > 20 mut/Mb; these results could not be replicated in bTMB < 20 mut/Mb [85,86].

Solange Peters presented POSEIDON clinical trial preliminary results at IASLC’s 2022 World Conference on Lung Cancer. Patients with metastatic NSCLC who received combined therapy of Tremelimumab, Durvalumab, and chemotherapy experienced higher 24-month OS compared to patients who received chemotherapy alone, regardless of STK11, KEAP1, or KRAS mutational status. This trial supports combining therapy as a potential first-line treatment option for these patients [87].

Another combination of therapies is Ipilimumab plus Pembrolizumab, which showed no benefit in advanced NSCLC. KEYNOTE-021 is a phase I/II trial that studied this combination at different dosing regimens. The primary endpoint of this trial was not met by the objective response rate of 30%, and no correlation was found with the PD-L1 tumor proportion score. The authors concluded that the combination had potential antitumor activity but with associated toxicity [88].

KEYNOTE 598 trial compared Pembrolizumab and Ipilimumab to Pembrolizumab alone in PD-L1 > 50% advanced NSCLC patients. PFS and OS were the primary endpoints in this case, but the trial was stopped due to no correlation with the better benefit of combination therapy over Pembrolizumab alone [89].

EMPOWER-lung 4 study is a phase II trial analyzing the efficacy of anti-PD-1 Cemiplimab alone at different doses or in combination with Ipilimumab. If treated with combination therapy, the primary endpoint ORR rate was 45.5% in PD-L1 1–50% and 36% in the PD-L1 negative population [90].

The Checkmate 012 study evaluated the different doses of Nivolumab and Ipilimumab in patients with recurrent stage IIIB or chemotherapy-naïve stage IV NSCLC [91]. Similar results were found in another phase III trial, checkmate 227. This study selected stage IV or recurrent NSCLC patients with PD-L1 > 1% and compared combination Nivolumab with Ipilimumab, Nivolumab monotherapy, and platinum-doublet chemotherapy alone. It showed an improvement in OS after Nivolumab plus Ipilimumab in both the PD-L1 positive and negative patients over chemotherapy alone, regardless of PD-L1 expression, TMB, or a combination of both biomarkers [92].

In 2020, the FDA approved this therapeutic strategy in PD-L1-positive advanced NSCLC as a first-line treatment [98]. Friedlaender et al. concluded that immunotherapy had become the first-line treatment for most patients with metastatic NSCLC and negative driver genes [99].

The same drugs were analyzed in a different regimen in Checkmate 9LA, a phase III trial that showed that the combination of Nivolumab and Ipilimumab with chemotherapy is associated with a significant improvement in overall survival in metastatic NSCLC compared to doublet chemotherapy alone. The benefit was seen in this study group regardless of the PD-L1 positivity and the histology (squamous and non-squamous) [93]. This quadruplet therapy has subsequently received FDA approval for this indication [100].

The combination of Nivolumab and Ipilimumab with or without chemotherapy is approved as the first-line strategy for PD-L1-positive NSCLC. However, it is still unclear the role of this therapy in metastatic NSCLC, especially considering the cost-effectiveness as described by Courtney et al. The authors concluded that to be cost-effective, immunotherapy should not exceed four months [101].

Despite the OS benefit of the combination immunotherapy demonstrated in the Checkmate 227 trial, this strategy is associated with an increased cost. Furthermore, treatment-related adverse events are more associated with adding Ipilimumab to chemotherapy or Nivolumab and chemotherapy [84,93].

Conversely, the Checkmate 9LA trial showed treatment-related adverse events in 47% of the cases treated with the quadruplet regimen compared to a 38% rate in the chemotherapy alone therapy [77]. Comparable results were found in the Ipilimumab plus chemotherapy (51%) versus chemotherapy alone (35%) [84].

Another treatment for metastatic NSCLC patients that have been studied is the combination of Ipilimumab with radiation. NCT02239900 is a phase I/II trial studying Ipilimumab and hypofractionated stereotactic radiation therapy (SBRT) as treatment in advanced NSCLC patients.

This trial evaluates the maximum dose tolerated by Ipilimumab associated with SBRT [94].

NCT03223155 evaluates the safety of concurrent or sequential Ipilimumab instead of Nivolumab and SBRT in patients with Stage IV NSCLC [95].

### 4.3. Metastatic NSCLC with Driver Mutations

Mazieres et al. reported a large retrospective study that demonstrated the poor clinical efficacy of immunotherapies in metastatic NSCLC patients with driver mutations. Especially in patients with EGFR or ALK mutations, the objective response rate was 12% and 0%, respectively, and the PFS were 2.1 and 2.5 months. For this reason, this therapy class has been abandoned in this patient population [79].

CTLA-4 inhibitors monotherapy or in combination with checkpoint inhibitors in metastatic NSCLC patients with targetable mutations have been evaluated only in very few studies and have been associated with adverse effects leading to early closure for safety [102].

One of these studies evaluated the combination of Ipilimumab with Erlotinib or Crizotinib as a first-line treatment strategy. Despite several adverse effects related to the treatment, such as colitis and leading to the early closure of this study, the long-term follow-up showed promising results with a median PFS of 17.9 and 21.4 months and a median OS of 42 and 47 months for 11 patients with EGFR and three patients with ALK mutation on trial, respectively. However, the reasons for these survival results are still not completely understood [103].

Considering these results, another phase I clinical trial evaluates the safety and efficacy of the combination of Osimertinib with Ipilimumab (NCT041141644).

Further clinical trials are studying the CTLA-4 inhibitor combinations in advanced EGFR/ALK mutation NSCLC patients with failed targeted therapy.

CheckMate 722 evaluates Nivolumab in addition to chemotherapy to Ipilimumab or chemotherapy alone in NSCLC patients with EGFR mutations who have failed first- or second-line EGFR TKI therapy (NCT02864251) [104]. The study results were presented by Mok et al. at the ESMO Asia Congress 2022 (Singapore, 2–4 December). They reported that adding a checkpoint inhibitor to chemotherapy in patients with NSCLC progressing on EGFR TKIs does not improve outcomes, and no significant improvement in PFS was reported [105].

In addition, another single-institution phase II study (NCT03256136) is evaluating Nivolumab plus chemotherapy, or Nivolumab in combination with Ipilimumab in EGFR or ALK mutated NSCLC patients who failed TKI therapy. Furthermore, NCT03091491 is a phase II study in Asia evaluating Nivolumab versus the combination of Nivolumab and Ipilimumab in a patient with EGFR mutation NSCLC who has failed first-line TKI therapy [106,107]. These three clinical trials are ongoing, and the results are pending (Table 6).

## 5. Conclusions

Induction immunotherapy with or without chemotherapy can be considered for unresectable locally advanced NSCLC, and after the downstaging, the possibility of surgery could be re-evaluated in a multidisciplinary setting with high rates of R0 resection. Furthermore, current data showed that lung resection for early-stage NSCLC followed by immunotherapy is safe and feasible.

Future research can help identify an objective biomarker to reliably predict the benefit of immunotherapy, the number of cycles and duration of immunotherapy in the preoperative or postoperative setting, and the optimal combination with other systemic therapies.

## Figures and Tables

**Table 1:** Clinical Trials: Neoadjuvant immunotherapy in resectable NSCLC.

Neoadjuvant Immunotherapy								
Trial/Study Name	Phase	Patient N	Neoadjuvant Therapy	Patient Population	Outcomes	Safety	MPR	PCR
NEOSTAR [20]	II	88	Nivolumab, Nivolumab ± Ipilimumab	IA-IIIA	Median OS and RFS not reached at 22.2 months	NR	22% vs. 38%	9% vs. 29%
LCMC3 [21]	II	181	Atezolizumab	IB-IIIB (resectable)	NR	NR	20%	7%
Gao et al. [22]	Ib	40	Sintilimab	IA-IIIB	R0 in 37/40	12.5% TRAEs grade 3–5	40.50%	16.20%
NEOMUN [23]	II	15	Pembrolizumab	II-IIIA	NR	33% TRAEs	13%	13%
PRINCEPS [24]	II	30	Atezolizumab	IA-IIIA	R0 in 29/30	3.3% TRAEs	14%	0%
IONESCO [25]	II	50	Durvalumab	IB (≥4 cm)-IIIA	R0 in 45/50	9% of death in 90 days	18.60%	7%
Tong et al. [26]	II	30	Pembrolizumab	IB-IIIA	R0 in 22/25	4% grade 3 TRAEs	28%	0%
Altorki et al. [27]	II	60	Durvalumab ± radiotherapy	IA-IIIA	R0 in 26/30 vs. 26/30	17% vs. 20% grade 3–4 TRAEs	6.7% vs. 26.6%	0% vs. 26.6%

MPR: Major Pathological Response, PCR: Pathological Complete Response. TRAEs: Treatment-Related Adverse Events, OS: Overall Survival, NR: Not Reported.

**Table 2:** Clinical Trials: Neoadjuvant Immuno-Chemotherapy in resectable NSCLC.

Neoadjuvant Immuno-Chemotherapy								
Trial/Study Name	Phase	Patient N	Neoadjuvant Therapy	Patient Population	Outcomes	Safety	MPR	PCR
NADIM [28]	II	46	Nivolumab + Carboplatin + Paclitaxel	IIIA	PFS 77%, OS 12–18-24 m: 97.8–93.5–89.9%	30% TRAEs grade 3–4	83%	71%
Shu et al. [29]	II	30	Atezolizumab + Carboplatin + nab-paclitaxel	IB-IIIA	R0 in 26/29 pts	50% TRAEs grade 3–4	57%	33%
NADIM II [30]	II	86	Paclitaxel + Carboplatin ± Nivolumab	IIIA-IIIB	Median OS 81.9% at 36 m	25% vs. 10.3% TRAEs grade 3–4	52.6% vs. 13.8%	36.8% vs. 6.9%
CheckMate 816 [31]	III	358	CT ± Nivolumab	IB-IIIA	R0 in 83% vs. 75%	11% vs. 15% TRAEs grade 3–4	37% vs. 9%	24% vs. 2%
KEYNOTE 671 [32]	III	NR	Pembrolizumab + CT	IIA-IIIA-IIIB (N2)	NR	NR	NR	NR
AEGEAN [33]	III	NR	CT ± Durvalumab	IIA-IIIA-IIIB (N2)	NR	NR	NR	NR
Checkmate 77T [20]	III	NR	CT ± Nivolumab	IIA-IIIB (T3N2)	NR	NR	NR	NR
IMPOWER 030 [34]	III	NR	CT ± Atezolizumab	II-IIIA-IIIB (T3N2)	NR	NR	NR	NR

MPR: Major Pathological Response, PCR: Pathological Complete Response. TRAEs: Treatment-Related Adverse Events, PFS: Progression-Free Survival, OS: Overall Survival, CT: Platinum-doublet chemotherapy, m: months, NR: Not Reported.

**Table 4:** Clinical Trials: Immuno-, Immuno-Chemo-, Immuno-Chemo-Radiotherapy in unresectable NSCLC.

Unresectable Stage III NSCLC								
Trial/Study Name	Phase	Patient N	Treatment Regimen	Patient Population	Outcomes	Safety	MPR	PCR
Van Reij et al. [54]	/	319	RT ± CT (Sequential vs. concurrent)	IIIA-IIIB	Seq: Median OS 17.4 m OS 5-y 17%, Con: Median OS 18.6 m OS 5-y 19%	NR	NR	NR
Bradley et al. [55]	III	544	Concurrent CT + High/low dose RT ± Cetuximab	IIIA-IIIB	High-dose RT Median OS 28.7 m, Standard-dose RT Median OS 20.3 m	NR	NR	NR
PACIFIC (Antonia et al.) 2017 [56]	III	713	Durvalumab vs. placebo	IIIA-IIIB	Median PFS 16.8 m vs. 5.6 m, Median time to death or distant metastasis 23.2 m vs. 14.6 m	29.9% vs. 26.1% TRAEs grade 3–4	NR	9% vs. 7%
PACIFIC (Spigel et al.) 2022 [57]	III	713	Durvalumab vs. placebo	IIIA-IIIB	Median OS 47.5 m vs. 29.1 m, Median PFS 16.9 m vs. 5.6 m	NR	NR	NR
PACIFIC-R [60]	III	1399	Durvalumab vs. placebo	IIIA-IIIB	Median PFS 21.7 m, Median OS NR	AESIs 27.7%	NR	NR
PACIFIC-6 [61]	II	117	Durvalumab	IIIA-IIIB	Median PFS 10.9 m, 12 m OS rate 84.1%	4% TRAEs	NR	0.80%
PACIFIC-2 [62]	III	300	Durvalumab	IIIA-IIIB	Not yet	Not yet	Not yet	Not yet
COAST [63]	II	189	Durvalumab ± Oleclumab or Monalizumab	IIIA-IIIB	Median PFS 6.3 with Durvalumab, NR Oleclumab, 15.1 m Monalizumab	TRAEs 39.4% with D, 40.7% with D + O, 27.9% with D + M	NR	3% with D, 1.7% with D + O, 4.8% with D + M
PACIFIC-9 [64]	II	/	Durvalumab ± Oleclumab or Monalizumab	IIIA-IIIB	Not yet	Not yet	Not yet	Not yet
CITYSCAPE [65]	II	135	Tiragolumab + Atezolizumab vs. Placebo	IIIA-IIIB	Median PFS 5.4 m vs. 3.6 m	TRAEs 12% vs. 3% grade 3–4–5	NR	NR
KEYNOTE 799 [67]	II	214	Pembrolizumab + cCRT + Paclitaxel + Carboplatin vs. Pembrolizumab + cCRT + Pemetrexed + Cisplatin	IIIA-IIIB	Not yet	TRAEs 64.3% vs. 51% grade 3–4–5	Not yet	Not yet
NICOLAS [69]	II	79	CRT ± Nivolumab	IIIA-IIIB	Median PFS 12.7 m, Median OS 38.8 m	TRAEs 9% vs. 18% grade 3–4–5	NR	NR
Checkmate 73L [71]	III	/	Nivolumab + cCRT + Nivolumab ± Ipilimumab vs. cCRT + Durvalumab	IIIA-IIIB	Not yet	Not yet	Not yet	Not yet
BTCRC-LUN16-081 [73]	II	105	cCRT + Nivolumab ± Ipilimumab	IIIA-IIIB	Not yet	TRAEs 32% vs. 44% grade 3–4	Not yet	Not yet
DUART [75]	II	150	RT ± Durvalumab	IIIA-IIIB	Not yet	Not yet	Not yet	Not yet
Mazieres et al. [76]	II	551	ICIs	IIIA-IIIB (with dGA)	Median OS 13.3 m, Median PFS 2.8 m	NR	NR	NR
Guisier et al. [77]	II	107	ICIs	IIIA-IIIB (BRAF-, HER2-, MET-, RET-)	Median OS 4.7 m, Median PFS 16.2	TRAEs 10% grade 3–4	Not yet	Not yet
Riudavets et al. [78]	II	323	CRT + Durvalumab	IIIA-IIIB	Median OS 47 m, Median PFS 17.5 m	TRAEs 6% grade 3–4, 0.5% grade 5	NR	NR

MPR: Major Pathological Response, PCR: Pathological Complete Response. TRAEs: Treatment-Related Adverse Events, PFS: Progression-Free Survival, OS: Overall Survival, CT: Chemotherapy, RT: Radiotherapy, cCRT: concurrent Chemo-Radiotherapy, D: Durvalumab, O: Oleclumab, M: Monalizumab, m: months, ICIs: Immune-checkpoint Inhibitors, dGA: driver Genic Alteration, NR: Not Reported.

**Table 5:** Clinical Trials: Immuno-, Immuno-Chemo-, Immuno-Chemo-radiotherapy in metastatic NSCLC without driver mutations.

Metastatic NSCLC without Driver Mutations						
Trial/Study Name	Phase	Patient N	Treatment Regimen	Patient Population	Outcomes	Safety
CheckMate 017 [79]	III	272	Nivolumab vs. Docetaxel	IIIB-IV squamous NSCLC	Median PFS: 3.5 m vs. 2.8 m. Median OS 9.2 m vs. 6.0 m	Any AE: 58% vs. 86%
CheckMate 057 [80]	III	582	Nivolumab vs. Docetaxel	IIIB-IV non-squamous NSCLC	Median PFS: 2.3 m vs. 4.2 m. Median OS 12.2 m vs. 9.4 m	Any AE: 69% vs. 88%
KEYNOTE-001 [81]	I	550	Pembrolizumab every 2 or 3 weeks	Locally advanced/metastatic NSCLC	Median OS 22.3 m, 10.5 m	TRAEs 12% vs. 6% grade 3–5
KEYNOTE-024 [82]	III	305	Pembrolizumab vs. CT	Advanced NSCLC	Median PFS 10.3 m vs. 6 m, Median OS 30 m vs. 14.2 m	Any AE: 73% vs. 90%
KEYNOTE-042 [83]	III	1274	Pembrolizumab vs. CT	Locally advanced/metastatic NSCLC (PD-L1 ≥ 50%, ≥20%, 1%)	Median PFS 7.1 m, 6.2 m, 5.4 m vs. 6.4 m, 6.6 m, 6.5 m. Median OS 20 m, 17.7 m, 16.7 m vs. 12.2 m, 13.0 m, 12.1 m	NR
Govindan R et al. [84]	III	956	Carboplatin + Paclitaxel ± Ipilimumab	IV or recurrent squamous NSCLC	Median PFS 5.6 m, 5.6 m. Median OS 13.4 m, 12.4 m	TRAEs 51% vs. 35% grade 3–4
NEPTUNE [85]	III	/	Durvalumab + Tremelimumab vs. CT	Metastatic	Not Yet	Not Yet
MYSTIC [86]	III	1118	Durvalumab ± Tremelimumab vs. CT	Metastatic	Median PFS 4.3 m, 3.9 m, 5.4 m; Median OS 16.3 m, 11.9 m, 12.9 m	Any AE: 54% vs. 60% vs. 83%
POSEIDON [87]	III	/	Durvalumab + CT ± Tremelimumab followed by Durvalumab ± Tremelimumab; vs. CT	Metastatic	Not Yet	Not Yet
KEYNOTE-021 [88]	I/II	44	Pembrolizumab ± Ipilimumab	Advanced NSCLC	Median PFS 4.1 m, Median OS 10.9 m	TRAEs 29% grade 3–5
KEYNOTE-598 [89]	III	568	Pembrolizumab ± Ipilimumab	Metastatic	Median PFS 8.2 m, 8.4 m. Median OS 21.4 m, 21.9 m	TRAEs 62.4% vs. 50.2% grade 3–5
EMPOWER-lung 4 [90]	II	28	Cemiplimab (3 weeks) ± Ipilimumab or Cemiplimab (108 weeks)	Advanced NSCLC	PD-L1 1–50%: ORR 45.5% PD-L1 <1%: ORR 36%	TRAEs 18.2%, 18.2%
Checkmate 012 [91]	I	78	Nivolumab + Ipilimumab (every 12 or 6 weeks)	Recurrent IIIB or IV	Median PFS 8.1 m, 3.9 m. Median OS NR	Any AE: 82%, 72%
Checkmate 227 [92]	III	1739	PD-L1 pos or neg: Nivolumab ± Ipilimumab vs. platinum-based CT	IV or recurrent	Median PFS: PD-L1 ≥1%: 5.1 m, 4.2 m, 5.6 m, PD-L1 <1%: 5.1 m, 5.6 m, 4.7 m; Median OS: PD-L1 ≥1%: 17.1 m, 15.7 m, 14.9 m, PD-L1 <1%: 17.2 m, 15.2 m, 12.2 m.	Any AE: 77%, 65.5, 84%, 76%, 92%, 78%
Checkmate 9LA [93]	III	719	Platinum-based CT ± Nivolumab + Ipilimumab	IV or recurrent	Median PFS 6.8 m, 5 m. Median OS 15.6 m, 10.9 m	Any AE: 91%, 87%
NCT02239900 [94]	I/II	35	Ipilimumab + SBRT	Advanced NSCLC	Median PFS 3.2 m, Median OS 10.2 m	TRAEs 34% grade 3
NCT03223155 [95]	I	78	Nivolumab/ipilimumab + sequential or concurrent SBRT	Metastatic	Not Yet	Not Yet

TRAEs: Treatment-Related Adverse Events, PFS: Progression-Free Survival, OS: Overall Survival, CT: Platinum-based chemotherapy, m: months, ORR: Overall Response Rate, AE: Adverse Effects, NR: Not Reported.

**Table 6:** Clinical Trials: Immuno-, Immuno-Chemo-, Immuno-Chemo-radiotherapy in metastatic NSCLC with driver mutations.

Metastatic NSCLC with Driver Mutations						
Trial/Study Name	Phase	Patient N	Treatment Regimen	Patient Population	Outcomes	Safety
Mazieres et al. [79]	II	551	ICIs	Metastatic NSCLC EGFR+ and ALK+	Median PFS 2.1 m, 2.5 m	NR
Chalmers AW et al. [103]	Ib	14	Ipilimumab + Erlotinib/Crizotinib	Advanced NSCLC EGFR+ and ALK+	Median PFS 17.9 m, 21.4 m; Median OS > 42 m, >47 m	TRAEs 78% vs. 33% grade 3
CheckMate 722 [104]	III	367	Nivolumab + Pemetrexed/CT or Nivolumab + Ipilimumab vs. Pemetrexed + CT	Metastatic or Recurrent NSCLC	Not Yet	Not Yet
Mok et al. [105]	III	294	Nivolumab + Platinum + Pemetrexed vs. CT alone	EGFR-mutated NSCLC	Median PFS 5.6 m, 5.4 m; Median OS 19.4 m, 15.9 m	NR
NCT03256136 [106]	II	9	Nivolumab + Carboplatin + Pemetrexed or Nivolumab + Ipilimumab	EGFR+ or ALK+	Not Yet	Not Yet
NCT03091491 [107]	II	31	Nivolumab ± Ipilimumab	EGFR+	Not Yet	Not Yet

TRAEs: Treatment-Related Adverse Events, PFS: Progression-Free Survival, OS: Overall Survival, CT: Platinum-based chemotherapy, m: months, ICIs: Immune Checkpoint Inhibitors, NR: Not Reported.

## Data Availability

The data underlying this article are available in the article.
